# The development of a sense of control scale

**DOI:** 10.3389/fpsyg.2015.01733

**Published:** 2015-11-06

**Authors:** Mia Y. Dong, Kristian Sandberg, Bo M. Bibby, Michael N. Pedersen, Morten Overgaard

**Affiliations:** ^1^Cognitive Neuroscience Research Unit, Center of Functionally Integrative Neuroscience, Aarhus University, Aarhus, Denmark; ^2^Hammel Neurorehabilitation and Research Centre, Aarhus University Hospital, Hammel, Denmark; ^3^Institute of Cognitive Neuroscience, University College London, London, UK; ^4^Department of Biostatistics, Aarhus University, Aarhus, Denmark

**Keywords:** sense of agency, sense of control, motor control, measuring scale, subjective experience, consciousness, awareness

## Abstract

In the past decades, sense of control—the feeling that one is in control of one’s actions has gained much scientific interests. Various scales have been used to measure sense of control in previous studies, yet no study has allowed participants to create a scale for rating their control experiences despite advances in the neighboring field of conscious vision has been linked to this approach. Here, we examined how participants preferred to rate sense of control during a simple motor control task by asking them to create a scale to be used to describe their sense of control experience during the task. Scale with six steps was most frequently created. Even though some variability was observed in the number of preferred scale steps, descriptions were highly similar across all participants when scales were converted to the same continuum. When we divided participants into groups based on their number of preferred scale steps, mean task performance and sense of control could be described as sigmoid functions of the noise level, and the function parameters were equivalent across groups. We also showed that task performance increased exponentially as a function of control rating, and that, again, function parameters were equivalent for all groups. In summary, the present study established a participant-generated 6-point sense of control rating scale for simple computerized motor control tasks that can be empirically tested against other measures of control in future studies.

## Introduction

In our daily life, we perform goal-directed actions and typically have a sense of being in control of those actions—of being the agent that performs the actions. As pointed out by Synofzik et al. (2008), this low-level subjective experience of being in control of an action is different from a higher order judgment of being an agent. This conscious sense of agency or sense of control has received much scientific interest over the last decades (Haggard et al., 2002; Metcalfe and Greene, 2007; David et al., 2008), yet the research is still ongoing and several aspects are still unresolved.

One aspect of general importance to the study of sense of control is how it is best measured. How can we know if we can trust the results of our experiments if we are not certain that the measures used to estimate the sense of control are optimal? Previous studies have used very different measures of the experience of control, but no studies have examined how participants prefer to report their control experience, and only very few have been concerned with scale generation. As an exception to this general rule, Polito et al. (2013) constructed a new measure for quantifying alterations in agency in a hypnotic state, which they termed the sense of agency rating scale (SOARS). The SOARS consisted of 10 items describing the experience of being an agent, and users were to rate each statement on a 7-point Likert scale (1 being “strongly disagree” to 7 being “strongly agree”). However, since SOARS was specifically developed to quantify agency in hypnotic context, it cannot be applied as a measure of sense of control in the majority of agency research. The general absence of sense of control scale literature is, in a sense, surprising given the amount of research conducted on participant constructed measures of awareness and comparison of measures of awareness in general in neighboring fields of consciousness research such as visual awareness (Ramsøy and Overgaard, 2004; Persaud et al., 2007; Persaud and McLeod, 2008; Sandberg et al., 2010, 2011) and artificial grammar knowledge (Persaud et al., 2007; Dienes and Seth, 2010; Wierzchoń et al., 2012).

Current experimental measures of sense of agency/control are highly diverse, yet two general approaches can be distinguished. These are implicit and explicit measures of sense of agency/control. Implicit measures of agency include intentional binding and the kinematics of a movement. The intentional binding paradigm captures how having a sense of agency affects the temporal relation between actions and effects. Haggard et al. (2002) proposed that the perceived timing of an external effect is shifted earlier if and only if the effect is preceded by a voluntary action with the intention to trigger such an effect. Kinematics of a movement reflects the relationship between the motor monitoring mechanism and an underlying action command that one cannot report verbally. In this sense, kinematics of movement can be used to demonstrate goal-directed behavior in the absence of awareness. For example, Fourneret and Jeannerod (1998) suggested that this type of sense of agency measure is able to capture the ability to adjust movement subtly in response to small deviations of an action outcome. This subtle adjustment in motor commands may otherwise go unnoticed by researchers because the person carrying out such an action may largely be unaware of them.

Explicit judgments of sense of agency, on the other hand, are typically assessed in paradigms using free or forced choice button presses or simple movements (rotation of a joystick, a finger tapping movement or following an object on the screen with the mouse) as the primary task. Following the primary task, participants are asked to indicate the degree to which they feel control over a certain action (Wegner et al., 2004; Linser and Goschke, 2007; Metcalfe and Greene, 2007) or to contribute a visual action effect to a particular agent, e.g., to themselves, the computer or another person (Aarts et al., 2005; Sato and Yasuda, 2005). Typically, these judgments are made on rating scales (10- or 100-point) ranging from “not me at all” to “definitely me,” or from “no control at all” to “complete control.” In other studies, participants are given pre-defined statements about their agency experience to rate using a Likert scale ranging from “strongly disagree” to “strongly agree” (five and seven steps being most common; Ma and Hommel, 2015). Another type of agency judgments typically involve self-other attribution, i.e., participants make “Yes” or “No” responses to indicate whether the images of a movement displayed on a computer screen reflects their own movement spatially and temporally (Farrer et al., 2003).

It is nevertheless difficult to judge which measure to use as scales have not been directly compared. It has been argued that a measure of conscious experience should be both exclusive and exhaustive (Reingold and Merikle, 1988). A measure is considered exclusive when it does not mistake unconscious processing for conscious processing. It could, for instance, be argued that if sense of control was estimated only by how well an action is performed then all accurate, but unattended and unconscious movements will be misclassified as reflecting a high sense of control. However, a good measure of awareness should not only avoid misclassifying unconscious processes as conscious, it should also ensure that all conscious processes are reported, or in the context of motor control that all feelings of control are reported. That is, it should be optimally exhaustive. An example of suboptimal exhaustiveness is when a measure fails to capture weak experiences and erroneously finds above-chance accuracy when no sense of control is reported, thus misclassifying a partially conscious movement as entirely unconscious.

For visual awareness, various measures have been compared including an introspective awareness measure (the perceptual awareness scale, PAS) generated by participants in a masked visual identification study (Ramsøy and Overgaard, 2004). PAS has been compared to more indirect measures of awareness—confidence ratings and post-decision wagering (Sandberg et al., 2010). The exhaustiveness of the measures was estimated using two common procedures. First, the task accuracy was estimated at the reports of no awareness, no confidence, or the lowest willingness to wager on being correct—i.e., the subjective threshold of each measure was established (Sidis, 1898). The scale with the lowest accuracy at the subjective threshold was considered to be most exhaustive—i.e., participants are willing/able to report even weak experiences. Second, the correlation between accuracy and awareness was estimated as a typical measure of awareness—i.e., the participants are not only willing to report awareness, but the awareness ratings are meaningfully related to accuracy. In the study, PAS performed better than the other two measures in both analyses, and additionally the awareness ratings were used more consistently across different stimulus durations (Sandberg et al., 2010). Some of the key findings of this study were recently replicated (Wierzchoń et al., 2014).

In the study presented below, our goal was to introduce and internally validate a similar participant-developed measure of sense of control for use in noisy movement tasks to be compared against other measures in future studies. Specifically, our participants each created a scale to describe their subjective sense of control over a goal-directed noisy mouse cursor movement, and the number of scale steps and the descriptions of control for each step was compared across participants. Subsequently for analyses, participants were divided into three groups based on the number of scale steps they preferred, and the influence of group on the accuracy-control relationship was examined. Overall, participants gave highly similar descriptions of scale steps, and using equivalence tests we found that the task performance (as measured by the Euclidean distance between final cursor position and target location in a pixel-based unit)—sense of control relationship was equivalent for all groups. For this reason, a single sense of control scale was created based on reports of all participants, what we have called the “SCS”. We believe this scale can be used in similar tasks, and the procedure for creating the scale may be used for scale creation in different experimental paradigms.

## Experiment 1

The main goal of Experiment 1 was to investigate how participants preferred to report their sense of control (specifically how many scale steps were preferred and how each scale step might be described), and whether the preferred scale had an impact on the performance-control relationship. To investigate this, participants were asked to construct a scale to measure their sense of motor control over a simple noisy mouse cursor movement.

### Methods

#### Participants

Thirty-five healthy participants [13 males; mean age 24 years (18–37); all right-handed] with normal or corrected-to-normal vision were recruited. All participants gave informed consent after reading an information sheet describing their rights as participants as well as the experiment, and they were debriefed afterward. During the debriefing, one participant expressed misunderstanding of the experiment (rating task difficulty rather than sense of control) and was subsequently removed from all analyses. Three other participants were excluded from the curve analysis due to insufficient number of trials with finalized rating scale (see Results, for a detailed description). Simple behavioral experiments such as the one reported here do not require ethical approval under Danish law, specifically Komitéloven §7 and §8.1.

#### Stimuli and Procedure

**FIGURE 1:**
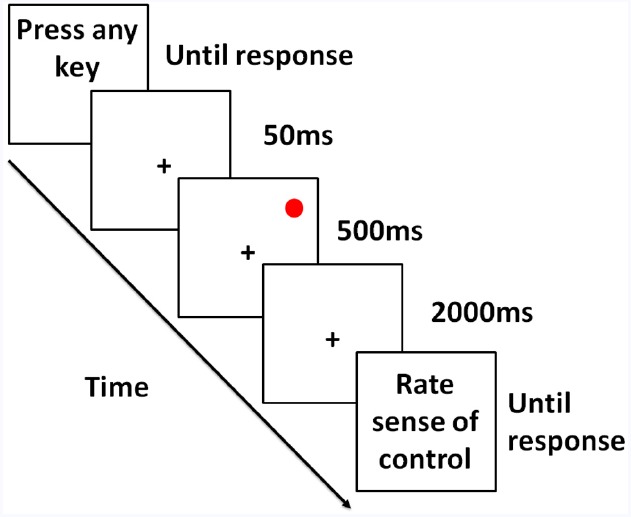
**Illustration of the motor movement paradigm.** After initiation of the trial, a fixation appeared briefly followed by a red target presented for 500 ms. After the target disappeared, participants were instructed to move the mouse cursor to the remembered target location within 2 s. Cursor movement was affected by varying levels of noise. At the end of each trial, participants were asked to write down their experience. Participants were instructed to create a scale to report experiences by the end of the first block (of 4) and update this scale over the course of the experiment if necessary.

Participants were seated at a desk facing a LED monitor with a display frequency of 60 Hz at a distance of approximately 60 cm. They performed a simple motor task (Figure 1). The background color was light gray with a luminance of 80% of screen maximum, and the target was a red dot [RGB value: (255, 0, 0)] with a size of approximately 0.2°of visual angle. On each trial, participants were asked to press any key when ready to start the trial. Next, a fixation cross appeared at the center of the screen for 50 ms followed by the target appearing at a random location on the screen for 500 ms. When the target disappeared, participants had 2000 ms to move a cursor from the center of the screen to the remembered target location using a mouse. Task performance was measured as the Euclidean distance between final cursor position and target location in a pixel-based unit.

On a given trial, one of two types of noise was added to the cursor movement. The purpose of using two noise types was to avoid ratings of sense of control being tied to one particular noise type. For both noise types, it was ensured that an increase in noise would have a detrimental effect on performance, and that performance would cover the entire possible range from floor (i.e., behavioral response has no impact on accuracy) to ceiling (participants are as accurate as they can be). For both noise types, the velocity of the cursor was affected, and the cursor bounced back upon hitting the edge of the screen. Noise was triggered by movement of the cursor by the participant and could influence cursor movement in any direction with a random delay (non-uniform) of 1–250 (mean = 16.67) milliseconds. For one type of noise, the movement of the cursor was affected linearly in one direction, and for the other type of noise, the movement of the cursor was affected by a sine wave movement toward one general direction. For each noise type, there were six levels of computer interference/noise ranging from no noise at all (0% interference, all participant input) to complete noise (cursor movement was completely driven by the computer and no participant input, 100% interference) in steps of 20%. At the end of each trial, participants were asked to evaluate how much control over the cursor movement they experienced using the guidelines described in the paragraph below. Finally, the participants initiated the next trial by pressing a button on the keyboard. All participants completed four blocks with 72 trials in each block, a total of 288 trials.

#### Scale Development

Participants were given written and verbal instructions on how to generate a scale to describe their experience of control over the cursor movement. They were informed that after each trial, they would be asked to evaluate how much control they experienced over the cursor movement they had just carried out. The end goal of the task was to construct a scale with which they were comfortable reporting their sense of control over cursor movement. Participants were suggested to perform this task in steps, e.g., first take down notes after each trial recording what type of experience it was, after they have an idea of what to expect in the task, they could start constructing a sketchy scale with scale points and corresponding descriptions. The end result scale should be a scale with as many points as necessary to describe their subjective experience but no more than needed. Participants were instructed to have a preliminary scale after the first block (72 trials) and update the number of scale steps and the scale step descriptions, if necessary, over the course of the remaining three blocks (216 trials).

#### Statistical Analyses

The data were analyzed using R version 3.1.2 (non-linear regression models) and Stata 14 (random coefficient models). Random coefficient models were used to estimate the relationship between control ratings and performance, and non-linear regression models were model performance and control ratings as sigmoid functions of noise level. To ensure comparability between the scales created by all participants, all scales were transformed to start at 1 and end at 6, which was the most commonly chosen number of scale points. The details of the analyses and the transformation are reported in the Appendix.

#### Equivalence Tests

As we were particularly interested in examining whether the relationship between the performance and the control ratings was similar across groups, equivalence tests were performed. In brief, these tests allow us to accept/reject whether a difference between two groups is within certain equivalence limits. An in-depth explanation of these tests and our chosen limits for the tests is reported in the Appendix.

### Results

#### Scale Point Distribution

**FIGURE 2:**
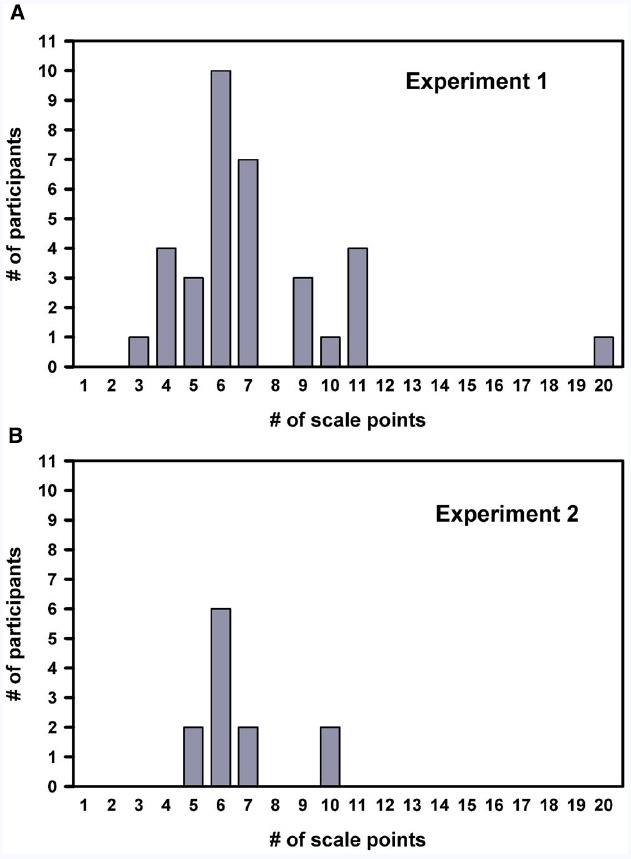
**Distribution of preferred number of scale steps.** Bar charts showing the distribution of the number of steps on the scales created by the 34 participants in Experiment 1 **(A)** and 12 participants in Experiment 2 **(B)**. Both distributions were well described by a log-normal distribution and had a mode of 6.

We first examined the distribution of the number of scale points participants constructed (i.e., how many scale steps participants found to be necessary and sufficient to report their experience). As seen in Figure 2A, the distribution was right skewed, and the number of scale points ranged from 3 to 20 with the majority finalizing their rating scale at 6 points (i.e., the mode was 6). Histogram inspection of log-transformed number of scale step data indicated that the distribution was log-normal. The geometric mean was 6.66 [95% CI (5.84; 7.60)]. Overall, the most frequently preferred scale thus consisted of six steps, but some variability was observed.

A main goal of the study was to create a single scale for use in similar experimental paradigms in future studies, yet participants spontaneously selected different numbers of scale points. In order to examine whether these scales were comparable and could meaningfully be collapsed to a single, most commonly preferred scale, we compared the relationship between accuracy in the motor task as a function of ratings of control for all participants: those preferring six steps, those preferring fewer, and those preferring more. This division resulted in three groups: group 1 consisted of seven participants who created rating scales with less than 6 rating points; group 2 consisted of 10 participants who created a 6-point scale, and group 3 consisted of 14 participants who created scales with more than 6-points to describe their subjective experience. While the majority of participants finalized their rating scale after the first block, leaving three blocks for analyses, three modified the number of steps in their scale throughout all four blocks. This gave insufficient number of blocks (0–1) after finalizing their scale for meaningful analyses, and they were therefore excluded from further analyses. In order to compare the relationship between task performance and subjective ratings across groups, we transformed all experience ratings to fit a scale ranging from 1 to 6 as described in the Section “Methods.”

#### Accuracy as a Function of Control Rating

**FIGURE 3:**
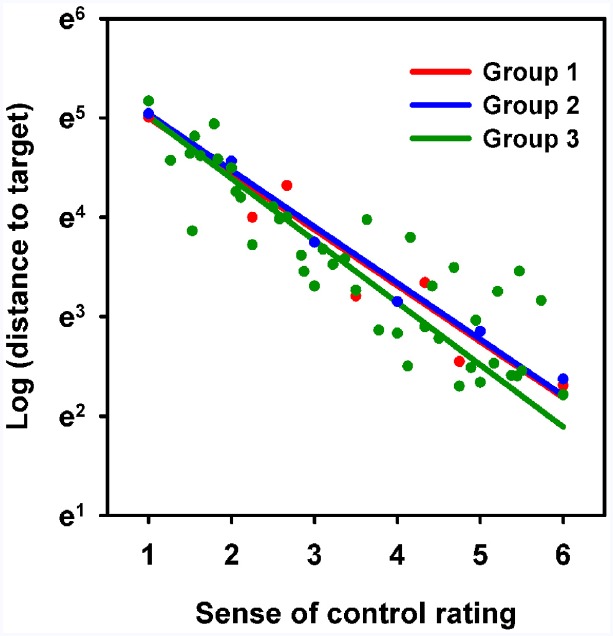
**Relationship between converted sense of control rating and distance to target.** Scatter plot showing mean distance to target as a function of the converted sense of control rating for each group (logarithmic y-axis). Lines represent random coefficient model fits for each group. Note that the linear fit implies that distance to target decreases exponentially as a function of sense of control rating.

The relationship between performance and sense of control rating was examined using random coefficient models. As the relationship appeared exponential, the distance to target data was log transformed, and Q–Q plot and residual plot inspection did not contradict this relationship. Log (distance to target) was modeled as a linear function of group (between-participant categorical variable) and the interaction between group and converted rating (within-participant continuous variable). The intercept was set at sense of control = 1. Participant number was modeled as a random effect. Interestingly, the functions for each group were highly similar, and no significant difference was found between groups [*χ*^2^(2) = 1.58, *p* = 0.45]. The resulting models for each group are plotted in Figure 3. The result was qualitatively unchanged when noise level was included in the model as an independent variable [*χ*^2^(2) = 0.88, *p* = 0.65]. For group 1, the geometric mean of the intercept value was 148 [95% CI: (125; 176)]. For group 2, the geometric mean of the intercept value was 154 [95% CI: (133; 179)]. For group 3, the geometric mean of the intercept value was 151 [95% CI: (133; 170)].

Equivalence tests were performed with the mean slope value (0.59) as the limit (see Methods). The intercept was 0.039 [90% CI: (–0.16; 0.24) lower for group 1 than for group 2, and the hypothesis of non-equivalence was rejected (*p* = 0.0001)]. The intercept was 0.016 [90% CI: (–0.17; 0.20) lower for group 1 than for group 3, and the hypothesis of non-equivalence was rejected (*p* < 0.0001)]. The intercept was 0.024 [90% CI: (–0.19; 0.14) higher for group 2 than for group 3, and the hypothesis of non-equivalence was rejected (*p* < 0.0001)].

For group 1, the slope was –0.56 [95% CI: (–0.66; –0.47)]. For group 2, the slope was –0.57 [95% CI: (–0.65; –0.48)]. For group 3, the slope was –0.62 [95% CI: (–0.69; –0.56)]. In other words, for group 1, an increase in sense of control rating of 1 corresponded to a decrease of 43% (37%; 48%) in distance to target. For group 2, the corresponding value was 43% (38%; 48%). For group 3, distance to target was reduced by 46% (43%; 50%) as sense of control increased by 1 rating step. Equivalence tests were performed with 50% of the mean slope value (0.59 × 0.5 = 0.295) as the limit (see Methods). The slope was 0.0038 [90% CI: (–0.10; 0.11)] larger for group 1 than for group 2, and the hypothesis of non-equivalence was rejected (*p* < 0.005). The slope was 0.062 [90% CI: (–0.038; 0.16)] larger for group 1 than for group 3, and the hypothesis of non-equivalence was rejected (*p* < 0.01). The slope was 0.058 [90% CI: (–0.032; 0.15)] larger for group 2 than for group 3, and the hypothesis of non-equivalence was rejected (*p* < 0.005).

In conclusion, the relationship between task performance and converted (i.e., relative) sense of control rating was described by highly similar exponential functions across groups, and tests with conservative limits found the models to be equivalent.

#### Curve Fitting (Non-Linear Regression)

**FIGURE 4:**
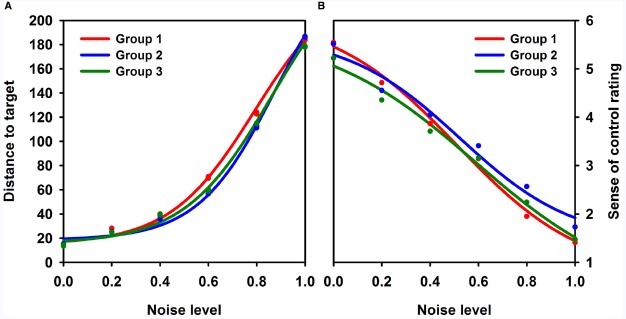
**Task performance and control rating as functions of noise level.** Task performance (distance to target in a pixel-based unit) **(A)** and sense of control rating **(B)** plotted as functions of noise level for all groups. Group 1 (black) consisted of participants using scales with fewer than six steps, group 2 consisted of participants using a 6-point scale, and group 3 consisted of participants using scales with more than 6-points. For groups 1 and 3, the ratings were transformed to fit an interval scale from 1 to 6 to ensure comparability between groups. Four-parameter sigmoid functions were fitted to the data.

**FIGURE A1:**
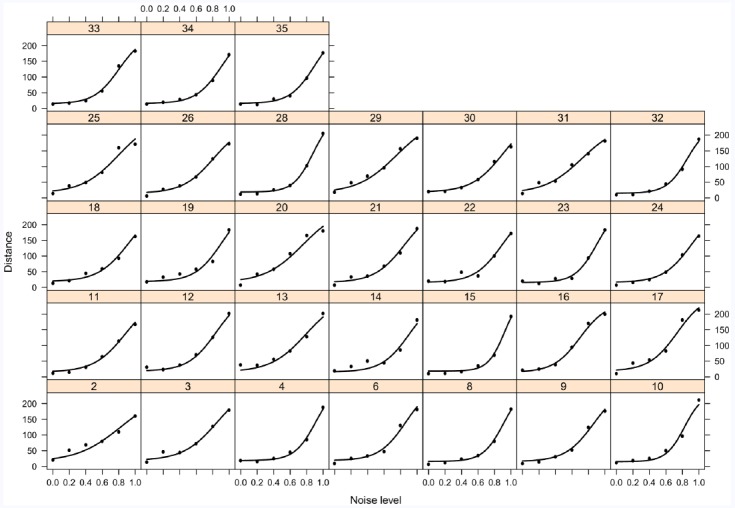
**Figure A1. Task performance (distance to target) plotted as functions of noise level for each participant.** Four-parameter sigmoid functions were fitted to the data.

**FIGURE A2:**
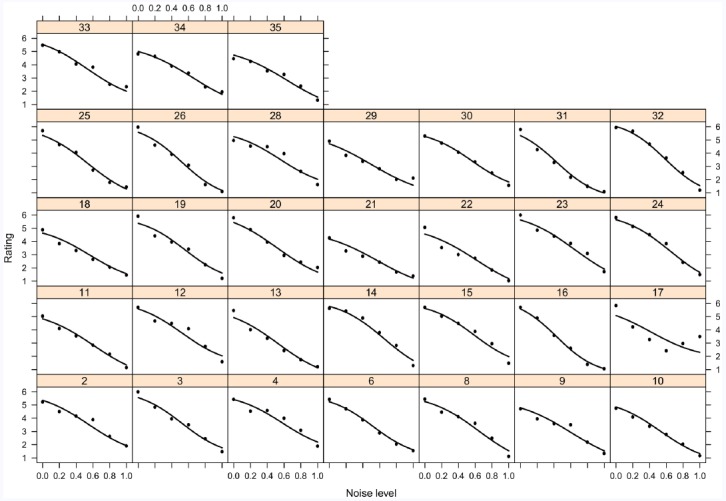
**Figure A2. Sense of control rating plotted as functions of noise level for each participant.** Four-parameter sigmoid functions were fitted to the data.

Four-parameter sigmoid functions were fitted to the distance-to-target data (the performance curve) and to the sense-of-control data (the rating curve) for each participant using the non-linear regression model described above. The parameters of the non-linear regression model are shown with 95% confidence intervals in Table 1, and the group curves are shown in Figure 4 (see Figures A1 and A2, for individual participant curves). Note that for all groups, mean distance to target and mean control rating develop meaningfully as a function of noise level (i.e., more noise leads to larger distance to target and lower sense of control).

**TABLE 1:** **Sigmoid function parameters**.

**Parameter**		***a***	***b***	***c***	***d***
Group 1	Performance	14.91 (8.12; 21.70)	235.31 (200.62; 270.00)	0.79 (0.71; 0.87)	0.18 (0.13; 0.22)
	Rating	6.16 (5.16; 7.16)	0.68 (–0.30; 1.67)	0.51 (0.41; 0.61)	0.27 (0.14; 0.40)
Group 2	Performance	18.56 (13.62; 23.51)	264.22 (216.18; 312.26)	0.87 (0.80; 0.95)	0.16 (0.12; 0.20)
	Rating	5.89 (5.00; 6.79)	1.13 (0.08; 2.90)	0.54 (0.43; 0.64)	0.28 (0.13; 0.43)
Group 3	Performance	15.68 (10.94; 20.43)	256.27 (214.05; 298.50)	0.86 (0.78; 0.93)	0.18 (0.14; 0.22)
	Rating	5.77 (4.93; 6.61)	0.31 (–0.98; 1.60)	0.60 (0.48; 0.71)	0.32 (0.17; 0.47)

The relationship between distance to target (performance) and sense of control (rating) were explained as sigmoid functions of noise level. The parameters for the functions are reported here with 95% confidence intervals.

No parameter differed statistically significantly between groups for either sense of control or task performance curves (*p* > 0.23 for all comparisons). As we were specifically interested in establishing that the differences in the c parameter did not show large variation between groups, equivalence tests were performed as described in the Methods. Specifically, we tested if the lag between performance and control curves varied by no more than 30% noise across groups. The difference in performance-control lag was 7.25% [90% CI: (–6.9; 21.4) smaller for group 1 than for group 2, and the hypothesis of non-equivalence was rejected (*p* = 0.006)]. The difference in performance-control lag was 0.50% [90% CI: (–15.0; 16.0) larger for group 1 than for group 3, and the hypothesis of non-equivalence was rejected (*p* = 0.002)]. The difference in performance-control lag was 7.75% [90% CI: (–7.6; 23.0) larger for group 2 than for group 3, and the hypothesis of non-equivalence was rejected (*p* = 0.01)].

Taken together, these results showed that the relationships between noise and task performance as well as sense of control ratings were highly similar for all groups, suggesting that subjective experience of control and task accuracy were comparable between groups when participants were allowed to use a scale they have constructed themselves. For this reason, analyses of scale step descriptions were performed for all participants as a whole.

#### Scale Description

**FIGURE 5:**
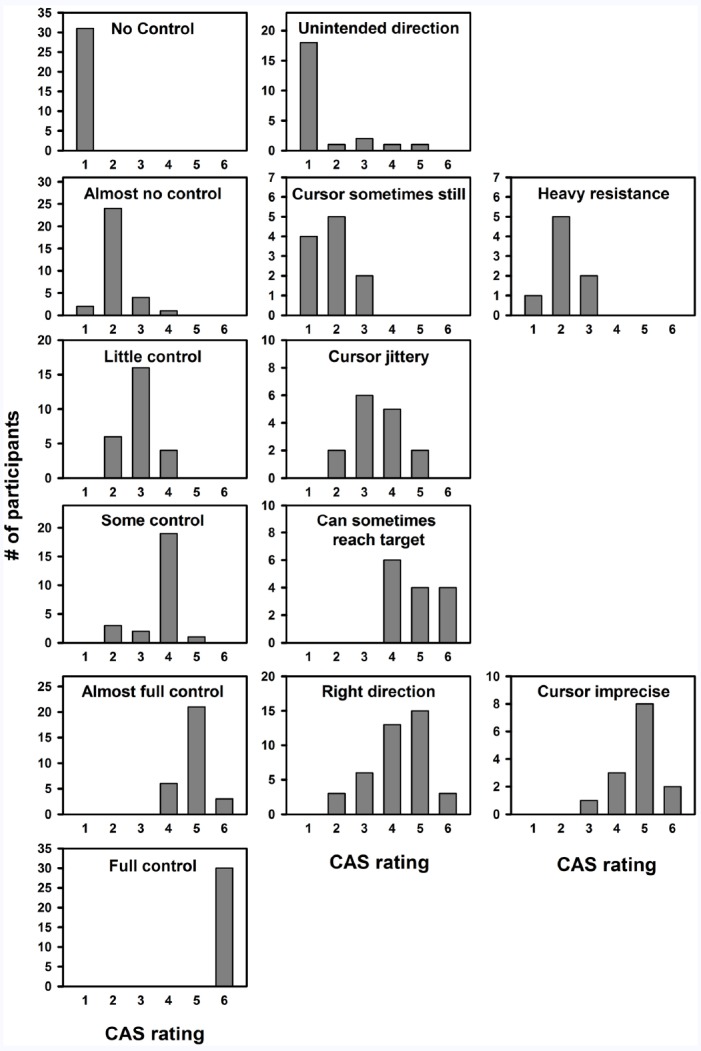
**Distribution of ratings for each description.** Plots show the number of participants using a certain description of their experience of control as a function of the mean control rating (e.g., 31 participants used the description “No control,” and they all used this description at scale step 1). Scales were converted to an interval scale from 1 to 6 for all participants and converted ratings were rounded up/down subsequently. Plots are sorted by mode (row 1 plots have a mode of 1, etc.).

We analyzed the descriptions associated with each scale step for two reasons: (1) It would allow us to examine if participants provide similar descriptions of their sense of control. (2) It would allow us to label each step and provide a short description of the associated sense of control. In order to examine the descriptions of participants’ sense of control the rating descriptions were broken down into short phrases using the key words such as “no control,” “the cursor moves in unintended direction,” or “I can initiate the cursor movement.” For each participant, descriptions were categorized by converted rating. As converted ratings were not always integers, they were rounded up/down when needed. The results are plotted in Figure 5.

### Interim Discussion and Conclusion

Experiment 1 demonstrated that when asked to describe their feeling of control over a simple noisy movement, a large part of participants preferred to use a 6-point scale, and the scale preference of the remaining participants were scattered around this value following a log-normal distribution. The experiment also showed that despite using different rating scales, the relationship between noise level and sense of control as well as that between noise level and task performance were consistent across participants. Similarly, the relationship between control rating and task performance was consistent across participants, and overall performance increased as a function of control rating. An increase in sense of control rating of 1 corresponded to, on average, a drop of 43–46% in distance to target for all three groups (i.e., the error distance was approximately halved for each increasing control rating). Finally, we found that participants used highly similar written descriptions of their sense of control and that they used these descriptions at similar levels of control. Overall, it appeared that participants spontaneously reported their sense of control in a highly similar manner, and we argue that this may form the basis of a SCS created on the basis of participant reports. We propose that such a SCS has six steps with the labels and descriptions provided in Table 2. It should be noted that we do not propose this scale as a universal measure of agency, but rather as specific to the general type of task employed here. We return to this topic of generalisability in the General Discussion.

**TABLE 2:** **Sense of control scale (SCS) including scale step descriptions**.

**SCS rating**	**Scale description**		
1	No control	Unintended direction	
2	Almost no control	Cursor sometimes still	Heavy resistance
3	Little control	Cursor jittery	
4	Some control	Can sometimes reach target	
5	Almost full control	Right direction	Cursor imprecise
6	Full control		

Note that the primary, most frequent descriptions (the first column of scale descriptions) appear to be somewhat general and directly related to the experience of control whereas the secondary, less common descriptions (the latter two column) appear to be more task specific and more often related to cursor accuracy.

It should also be noted that in the experiment used to create this scale, exactly six different noise levels were used, and one reason for observing a 6-point SCS may have been that participants were able to detect the six level graded changes in computer interference in the experimental setting. Therefore, we carried out a control experiment to examine whether this was the case.

## Experiment 2

The main goal of this experiment was to examine whether participants’ preference for a 6-point SCS was independent of the number of noise levels in the behavioral task. In this experiment, we asked a new group of participants to perform a behavioral task identical to that of Experiment 1, except that 36 noise levels were used instead of 6.

### Methods

#### Participants

A new cohort of 12 healthy participants (2 males; 19–26 years; mean age 23; all right-handed) with normal or corrected-to-normal vision participated. All participants gave informed consent after reading an information sheet that states their rights as participants and experiment information. They were also debriefed afterward.

#### Stimuli and Procedure

The stimuli were identical to those in Experiment 1, except that 36 evenly spaced noise steps ranging from no noise at all to complete noise were used. The total number of trials (288) was identical to that used in Experiment 1 thus resulting in eight trials per noise level versus 48 in Experiment 1.

### Results

#### Scale Distribution

We examined the distribution of the number of scale points participants constructed in Experiment 2. As seen in Figure 2B, the distribution was right-skewed, and scale points ranged from 5 to 10. As in Experiment 1, the mode was 6. The number of scale steps was log-transformed, and histogram inspection indicated that the distribution was log-normal. The geometric mean was 6.40 [95% CI (5.51; 7.45)], which did not differ significantly [*t*(46) = 0.34, *p* = 0.73] from the mean observed in Experiment 1 [6.66, 95% CI (5.84; 7.60)].

### Interim Discussion and Conclusion

As in Experiment 1, participants in Experiment 2 typically preferred reporting their sense of control on a 6-point rating scale although they were exposed to 36 different noise levels throughout the experiment. The number of scale steps thus appears unaffected by the number of noise levels used in the experiment.

## General Discussion

In this study, we set out to test whether it is possible to create an explicit (or direct) measure of the subjective sense of control in a noisy movement task. Within the framework of a specific experimental paradigm, we believe to have done so using a very similar procedure as what has previously been done in visual consciousness research (Sandberg and Overgaard, 2015). Two experiments were conducted, and, taken together, they showed that despite observing variability in the number of scale steps created, participants typically preferred a 6-point rating scale when reporting their sense of control. Experiment 1 further demonstrated that the relationship between noise level and sense of control, as well as noise level and task performance was highly comparable regardless of how many scale points were developed. Consistent with this finding, participants also assigned highly similar descriptions to the scale steps. On this basis, it seems plausible to suggest that there is something generalizable, shared between individuals, to this subjective experience of having control that can be introspectively accessed, described, and quantitatively measured.

This may be surprising as previous studies comparing awareness scales (e.g., in vision research) have found that the relationship between accuracy and awareness varies across scales, and in particular that ratings of “no awareness” (or similar) do not have the same meaning on different scales—demonstrating that the scales indicate different degrees of unconscious processing by the subjective threshold approach. For example, one experiment compared the PAS, with a dichotomous scale (“not seen”/“seen”) and found that accuracy was higher for the “not seen” rating than for a PAS rating of 1 (“No experience”, thus indicating more subliminal perception; Overgaard et al., 2006). Similarly, when using the same “not seen”/“seen” scale, a patient fulfilled the criteria for blindsight, but when using PAS, performance as a function of awareness rating in her blind visual field followed the same pattern as in her healthy field (Overgaard et al., 2008). In contrast, Tunney and Shanks (2003) found that a binary “high”/“low” confidence scale was able to detect differences between conscious and unconscious processing in a very difficult task (55% accuracy, chance = 50%), whereas a continuous scale from 50 to 100 (indicating the estimated accuracy from chance to complete certainty) was not. Dienes (2008) found that when an easy task was used, the opposite pattern was observed. In contrast to these previous studies, our results show that when participants generate their own SCS, the criteria for each meaningful label (e.g., “no control”) does not vary with the number of scale steps. This has previously been emphasized as a general requirement for collapsing participant-generated scales with different numbers of scale steps into a single general scale (Sandberg and Overgaard, 2015).

Nevertheless, it should be noted that the generalisability of the proposed SCS, across experimental paradigms has not been tested in the present study—only the generalisability of the performance-sense of control relationship across participants. We have previously argued that scales for obtaining reports of subjective experience generated for one specific context may not be the optimal scale for another context, and if one needs the optimal scale for a given paradigm, a similar method as presented here and elsewhere may be used for scale generation (Sandberg et al., 2013; Andersen et al., 2015). Nevertheless, it should be noted that scales created in a particular experimental context generally work well in similar experiments (see Sandberg and Overgaard, 2015, for an overview). For this reason, SCS might fill a gap in current motor control research—the lack of an explicit measuring scale to investigate the conscious sense of control. It is also interesting on the basis that the approach used to develop SCS resulted in consistent correlations between subjective report and objective accuracy.

It may be noted that all participants spontaneously developed scales containing at least three rating steps and resulted in a gradual SCS rather than a dichotomous rating scale. This was the case even though participants were instructed to use no more scale points than needed to describe their control experience. This finding may be relevant to the debate of whether consciousness is a graded or dichotomous phenomenon (Sergent and Dehaene, 2004; Overgaard et al., 2006; Kouider et al., 2010; see Bachmann, 2000, for a historical account advocating the gradual stance).

One potential issue in sense of agency research that is also present in the current task design is that subjective measures do not distinguish between the evaluation of subjective, bodily experience and judgments of task performance. The current paradigm provided indirect feedback in that the moving cursor was visible to the participant at any point on the trajectory. In the literature, even more direct feedback has been given. For example, in Miele et al. (2011), the motor task involved catching “X”s falling from the top of the screen and avoid the “O”s by moving a bar across the computer screen. Different types of noises were introduced on the falling letters in order to manipulate the amount of control that participants experience. “X”s and “O”s disappeared upon being touched by the bar but would continue falling below the bar, thus providing visual feedback on performance. In addition, auditory feedback was also present, i.e., a “ping” sound was played each time an “X” was hit and a “pong” sound when an “O” was touched.

It is an open question exactly how great the impact of feedback is on ratings of control, but it is important to remember that even if some influence is present, judgments of task performance can be distinguished from reports of experience conceptually, behaviorally and neurally. In vision research, for example, participants are able to distinguish and switch between reporting the subjective clarity of perception (which may be viewed as the visual counterpart of sense of control) and confidence in being correct (which is based on evaluation of task performance; Sandberg et al., 2010; Zehetleitner and Rausch, 2013). Although the two types of reports are correlated, and they both correlate with objective performance, they are not identical, and important differences are observed (Sandberg et al., 2010). Similarly, although a strong correlation between sense of agency/control and behavioral task performance has been reported (Metcalfe and Greene, 2007; Metcalfe et al., 2010), neural correlates of the two types of judgments have been dissociated. Miele et al. (2011) observed that when participants made judgment of the amount of control they experienced, activation in the left anterior prefrontal cortex and right orbitofrontal cortex was increased compared to when they made judgment of performance. Taken together, we thus believe there are multiple reasons to believe that the participants in our study did not simply report their perceived accuracy. In addition, as the ratings of control were predictive of accuracy even when taking into account the physical noise level, the participants cannot simply have been reporting the observed noise either.

In spite of rating scales having received much attention in the last decade in visual consciousness and implicit learning research, the topic has only recently started to receive attention in the study of sense of control/agency as mentioned in the Introduction. In addition to the scale generation study mentioned in the introduction (Polito et al., 2013), a few studies have also recently been published on the comparison of measures of sense of control. For instance, one study (Ebert and Wegner, 2010) empirically examined the relationship between implicit and explicit measures of agency in action monitoring task. While implicit agency was assessed on a 10-point interval estimate scale, a 7-point scale that evaluates the extent to which one’s action caused the perceived effect examined explicit authorship attribution. Correlation between implicit and explicit measures was observed when the two questions were asked in the same block but not in different blocks, suggesting that these two agency questions may interfere with each other when assessed simultaneously.

Therefore, Saito et al. (2015) examined the two levels of agency in separate tasks and reported discrepancies between these two ways of quantifying sense of agency. The study was based on the assumption that there are two steps in agency judgment—first-level feeling of being an agent (which is tackled by implicit agency measures) and second-level judgment of agency (which is reflected by explicit judgment of self-other attribution). Saito and colleagues investigated these two levels of agency by assessing both implicit and explicit agency measures in the same population but with different tasks. A classical intentional binding task was employed as the implicit agency task, which was assumed to reflect participants’ ability in action regulation and perceptual processing—aspects in feeling of agency. Subsequently, an action monitoring task was carried out, and explicit judgment of agency was assessed by a “Yes”/“No” self-other attribution question. No significant correlation was found between the amount of binding in the implicit task and explicit measure of agency, leaving several interpretations open. The result may suggest that these two types of agency measures reflect different agency systems, or that a dichotomous self-other attribution question is too imprecise.

Overall, the examination and comparison of measures of agency appears to have just started within the field of motor control. In the neighboring field of visual awareness, a similar scale to SCS was created by (Ramsøy and Overgaard, 2004), namely, the PAS. In the original study, a positive correlation between awareness and accuracy was demonstrated, and as mentioned in the Introduction, a later study found that the PAS had advantages over other rating scales (Sandberg et al., 2010). Additionally, a recent study (Sandberg et al., 2014) compared PAS to an implicit measure of consciousness, a so-called exclusion task, and found that PAS might be more exhaustive as results indicated residual awareness on trials for which exclusion tasks indicated unconscious processing. In addition, use of the scale even indicated the presence of meaningful experiences in a blindsight patient (Overgaard et al., 2008). In the present study, we used similar experimental methods as was used in the Ramsøy and Overgaard (2004) study, but with a much larger sample size (5 in their main experiment vs. 47 across two experiments here). Also, data were analyzed using methods similar to the ones used in the Ramsøy and Overgaard (2004) study as well as more recent methods for comparing performance and awareness as functions of task difficulty (Sandberg et al., 2011).

It should be noted, however, that this study does not compare the SCS created here to alternative rating scales. As elaborated in the Introduction, the most appropriate measuring scale should be both exclusive and exhaustive. A logical step for future studies would thus be to compare explicit measuring scales such as the 6-point SCS to the dichotomous self-other attribution scale (“Yes” or “No”), confidence ratings as well as indirect measures such as intentional binding. Also, SCS may be further tested, refined and developed by applying it to a variety of different paradigms and tasks. Although more work needs to be done in order to propose a truly generalizable scale to measure the sense of control, we have presented an approach and demonstrated that one can compare the subjective experience of control between participants while generating meaningful data from subjective reports.

### Conflict of Interest Statement

The authors declare that the research was conducted in the absence of any commercial or financial relationships that could be construed as a potential conflict of interest.

## Appendix

This appendix contains a detailed description of the statistical analyses, including the performed equivalence tests, as well as plots of individual participant data. Figures A1 and A2 show that the sigmoid functions generally describe the individual data well. It may be noted that the sigmoid shape is less visible for the performance curve than for the control curve. This is because it is not as close to its theoretical maximum. From a modeling perspective, this is nevertheless not important as *a* and *b* parameters do not differ significantly across groups for both curve types.

### Statistical Analyses

First, we examined the distribution of the number of scale points participants constructed. The distribution was log-normal, and the mode of the distribution (i.e., 6) was selected as the number of scale steps to be used in further analyses (see Results, for details). Ratings on all other scales were transformed to fit a scale with a minimum of 1 and a maximum of 6 using a conversion based on relative distance to maximum and minimum. This was done using the equation: (*a* – 1)/(*b* – 1) × 5 + 1, where *a* corresponded to the scale point to be converted and *b* was the total number of scale points. For example, a rating of 4 on an 11-point scale would be converted to 2.5 on an interval scale from 1 to 6. Following scale conversion, participants were divided into three groups depending on the number of scale steps they had created. Group 1 included the participants using fewer than six steps, group 2 consisted of participants using exactly six steps, and group 3 included participants using more than six steps. For all group-based analyses, only blocks following finalization of the number of scale steps were used for each participant (the first block was typically discarded and only the last three blocks were included). Based on visual inspection, all three groups appeared to behave highly similarly within each type of noise so data from the two noise types was collapsed for all analyses to increase the statistical power for the more theoretically interesting question of whether participants in different groups behaved differently in general.

To examine if any difference in scale use was present across groups, two analyses were performed. First, the relationship between task performance (measured as the Euclidean distance between final cursor position and target location in a pixel-based unit) and sense of control ratings was estimated using a random coefficient model. Log-transformed distance to target was the dependent variable while group and converted rating were independent variables also adjusting for noise level. This corresponds to a linear regression model with group dependent slope and intercept, taking into account that the two measurements from the same participant are positively correlated. Noise level was adjusted for by including it as an additive continuous explanatory variable.

Second, comparison of psychometric curves for task performance and sense of control was performed using non-linear regression. Estimation of awareness through comparison of non-linear models of task accuracy and awareness has been used in multiple studies (Sandberg et al., 2011; Windey et al., 2013; Zehetleitner and Rausch, 2013) since it was proposed by Koch and Preuschoff (2007). Here, we used such non-linear regression models to describe the data. We estimated the lag differences between curves from different groups by comparing curve parameters. From a theoretical point of view, sigmoid functions were expected, and the sigmoid shape was identified by inspecting individual plots of the data. We chose the 4-parameter sigmoid function for all the analyses because it gave a good fit to all individual data. Furthermore, the function is very flexible and its parameters all have interpretations relevant to the problem at hand.

The 4-parameter sigmoid function is given by the expression *a* + (*b* – *a*)/(1 + *e*^(*c* – *x*)/*d*^) where *x* denotes the noise level. *a* denotes the plateau at small noise levels and *b* denotes the plateau at high noise levels. *c* denotes the *x*-value of the centre point of the slope (i.e., the point that is halfway between *a* and *b*). *d* is a measure of the steepness of the slope. Using this model, the lag between task performance and sense of control can be based on the difference in the values of the c parameter between the two functions. This is the main parameter of interest here. The absolute values of *a* and *b* are not important as both *c* and *d* are estimated on the basis of x-axis values corresponding to proportional changes of values on the y-axis, i.e., *c* is defined as the *x*-value that corresponds to the *y*-value that is exactly half of the difference between *a* and *b*. This way, comparisons between the *c* and/or *d* values can be made between a performance curve and a sense of control curve without the two curves sharing the same *a* and/or *b* value.

The parameters describing the sigmoid function were estimated using non-linear mixed effects regression analysis. More precisely, for each of the parameters *a*, *b*, *c*, and *d*, an interaction between group and response (distance to target/sense of control) was included as a fixed effect along with random effects corresponding to participant and the interaction between participant and response.

### Equivalence Tests

In order to conclude that the results (given by some parameter) for two groups are equivalent, equivalence limits are necessary.

If the true difference between the two groups (with respect to this parameter) lies within the limits, we say that the two groups are equivalent. An equivalence test is then performed as two one-sided tests of the difference being equal to each of the equivalence limits, and the corresponding *p*-value is the largest of the *p*-values from the one-sided tests. From an acceptance/rejection point of view this procedure is identical to constructing the 90% confidence interval for the group difference and reject non-equivalence (accept equivalence) if this interval is fully contained within the equivalence limits. In other words, if the 90% confidence interval is fully contained within the interval defined by the two equivalence limits, both one-sided test will be significant, and equivalence is accepted.

For analyses based on the random effects coefficient model, two equivalence tests were performed. First, we examined whether the intercepts were equivalent. The intercept corresponded to distance to target for the lowest control rating (1) and thus indicated the distance to target in the absence of control, a common measure of unconscious performance. It should be noted that we did not examine whether any unconscious performance was present as such (since there was no natural chance performance on our distance to target measure), but simply whether it was within the same range across groups (even if this range included no unconscious performance). We set the limit for accepting equivalence to a difference in distance to target corresponding to no more than what would be expected from an increase in control of one step on the SCS. This corresponded to a value of 0.59 for the log-transformed data, meaning that if the 90% confidence interval of the difference in intercept across groups did not include 0.59, we accepted equivalence (see Results, for details).

Second, we examined slope differences. The slope could in principle be any value from minus to plus infinity, but if control ratings are meaningful a negative value is expected (i.e., as control increases, distance to target is expected to be reduced). We set the limit for the equivalence test to a value to 50% of the slope value. With a slope of –0.59, the 90% confidence interval of the difference in slope across groups should not include 0.295 if we were to accept equivalence. Given the sample size and individual variability, the limits mentioned here corresponded to mean differences across groups of no more than a few percent of the examined values, and it may thus be considered relatively conservative limits.

For non-linear regression analyses, the control curve was expected to lag behind the performance curve (i.e., *a* difference in *c* parameters were expected), and this lag has been interpreted as an indication of subliminal processing (as control/awareness ratings increase slower than performance). In visual awareness experiments, this lag (i.e., *c* parameter difference) has been observed to correspond roughly to the value of d parameter of the awareness curve (corresponding to the control curve here; Sandberg et al., 2011). In other words, if the steepness parameter of a sigmoid function of subjective awareness reports of visual clarity as a function of stimulus presentation time in milliseconds has a value of 20 (ms), this curve is typically found to lag behind the identification performance curve by 20 ms. In the present study, the d parameter of all sense of control curves was around 0.30 (30% points noise difference), and this was chosen as the limit for our equivalence tests for the c parameter, meaning that if the 90% confidence interval of the difference in control-performance lag across groups did not include 0.3, we accepted equivalence. In other words, we tested if the observed differences in accuracy-control lag across groups were statistically significantly below 0.3 (i.e., 30% noise). Given the sample size and individual variability, this limit corresponded to mean lag differences across groups of no more than a few percentage points noise, and it may thus also be considered a relatively conservative limit.
